# Ultrasound Standing Wave-Based Cell-to-liquid Separation for Measuring Viscosity and Aggregation of Blood Sample

**DOI:** 10.3390/s20082284

**Published:** 2020-04-17

**Authors:** Gwangho Kim, Sanghwa Jeong, Yang Jun Kang

**Affiliations:** 1 PROTECHKOREA, 120-3 Nanosandan-ro, Samtae-ri, Nam-myeon, Jangseong-gun, Jeollanam-do 57248, Korea; gwangho.kim@daum.net; 2 Department of Mechanical Engineering, Chosun University, 309 Pilmun-daero, Dong-gu, Gwangju 61452, Korea

**Keywords:** ultrasonic transducer, RBCs separation, microfluidic device, blood viscosity, RBCs aggregation

## Abstract

When quantifying mechanical properties of blood samples flowing in closed fluidic circuits, blood samples are collected at specific intervals. Centrifugal separation is considered as a required procedure for preparing blood samples. However, the use of centrifuge is associated with several issues, including the potential for red blood cell (RBC) lysis, clotting activation, and RBC adhesions in the tube. In this study, an ultrasonic transducer is employed to separate RBCs or diluent from blood sample. The ultrasonic radiation force is much smaller than the centrifugal force acting in centrifuge, it can avoid critical issues occurring under centrifuge. Then, the RBC aggregation and blood viscosity of the blood sample are obtained using the microfluidic technique. According to the numerical results, ultrasonic transducers exhibited a maximum quality factor at an excitation frequency of 2.1 MHz. Periodic pattern of acoustic pressure fields were visualized experimentally as a column mode. The half wavelength obtained was as 0.5 λ = 0.378 ± 0.07 mm. The experimental results agreed with the analytical estimation sufficiently. An acoustic power of 2 W was selected carefully for separating RBCs or diluent from various blood samples (i.e., *Hct* = 20% ~ 50%; diluent: plasma, 1x phosphate-buffered saline (PBS), and dextran solution). The present method was employed to separate fixed blood samples which tended to stack inside the tube while using the centrifuge. Fixed RBCs were collected easily with an ultrasonic transducer. After various fixed blood samples with different base solutions (i.e., glutaraldehyde solution, 1x PBS, and dextran solution) were prepared using the present method, RBC aggregation and the viscosity of the blood sample are successfully obtained. In the near future, the present method will be integrated into ex vivo or in vitro fluidic circuit for measuring multiple mechanical properties of blood samples for a certain longer period.

## 1. Introduction

To monitor the mechanical properties of blood samples flowing under an extracorporeal rat bypass loop [1,2], blood samples are collected at specific intervals. The repetitive collection of blood sample results in decreasing the volume of blood flowing in the circuit substantially. To maintain a fixed blood volume, 1x phosphate-buffered saline (PBS) is infused. The hemodilution procedure leads to significantly change mechanical properties of blood samples (i.e., blood viscosity, hematocrit, and pressure) [3]. In addition, experimental tests have been limited to durations less than 2 h. The use of different blood storage techniques have led to large variations in the mechanical properties of the blood [4,5]. Among these properties, blood viscosity has been used extensively to evaluate the variations in blood samples, and it has been influenced by several factors, including hematocrit, red blood cells (RBCs) aggregation, RBC deformability, and plasma viscosity. As an important step, a centrifuge is used to separate components (i.e., cells or liquid) from the blood sample. After setting the hematocrit [*Hct*] to a specific level [6], the blood viscosity is measured with a viscometer. During the centrifuge operation, a strong centrifugal force acts on the blood cells. Fixed RBCs stack and adhere to the surface of the tube because of the strong centrifugal forces. The use of a centrifuge has led to several issues, including RBC lysis, clotting activation, and inflammatory cascades [7]. For the reason, it is difficult to collect fixed RBCs from the tube. Thus, it hinders in adding fixed RBCs into specific diluent (i.e., 1x PBS, plasma, and dextran solution). Our previous studies do not study the effect of diluents on fixed RBCs [8,9,10]. To resolve critical issues (i.e., periodic blood collection and blood separation with a centrifuge), it is necessary to separate RBCs or plasma from blood samples without using a centrifuge. Recently, several separation systems that utilize acoustic standing waves have been proposed to separate serum, lipids, platelets, and exosome from blood samples [11,12,13,14,15,16]. According to previous studies, ultrasonic standing pressure fields are employed to separate RBCs flowing in microfluidic channels. Acoustic radial forces contribute to migrating RBCs laterally along the flow streams. The ultrasonic transducer has a much smaller scale of acoustic radiation force when compared with a centrifugal force. Thus, it has been considered as a promising method that can resolve critical issues related to the use of a centrifuge. However, the methods are not effective to separate large volume of blood sample (~ 5 mL). Additionally, most previous researches only focus on cell separation of blood samples.

In this study, instead of a centrifuge, a bulk-sized ultrasonic transducer is suggested and designed to accelerate sedimentation of RBCs in stationary column chamber. To effectively separate RBCs or diluent from blood sample, the performance validation of ultrasonic transducer is validated by means of numerical analysis and experimental demonstration. First, numerical simulations are used to select optimum excitation frequency of acoustic standing wave by evaluating the resonance frequency, acoustic pressure fields, and quality factor. Second, after filling the ultrasonic transducer with the blood sample (*Hct* = 2%), focusing and sedimentation of RBCs inside the transducer are visualized experimentally with an elapse of time. Third, the contributions of the acoustic power and blood samples (i.e., hematocrit or base solution) to the separation performance are experimentally examined by measuring the separation height (*Hs*) over time. Fourth, variations of *Hs* are monitored by changing the degree of the RBC hardness or diluents. After separating RBCs from blood samples using an ultrasonic transducer, the blood sample is adjusted to a specific level of hematocrit by adding RBCs to a specific concentration of diluent. A microfluidic technique is employed to evaluate the mechanical properties of the blood samples (i.e., RBC aggregation and blood viscosity) with respect to the fixed RBCs or diluents of the blood sample.

## 2. Materials and Methods

### 2.1. Cell-to-Liquid Separation with Ultrasonic Transducer

**Figure 1:**
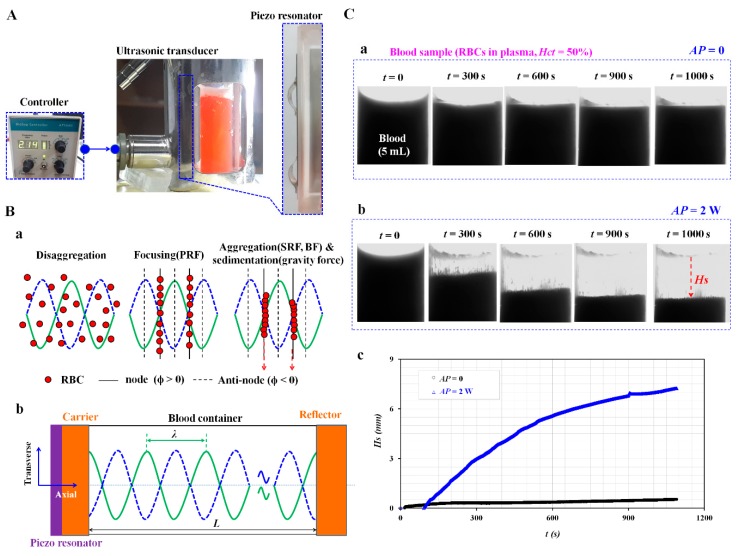
Proposed method for separating RBCs from blood sample with ultrasonic transducer. (**A**) Ultrasonic experimental setup, including an ultrasonic transducer and a controller. Right side panel showed piezo resonator in ultrasonic transducer. (**B**) The working principle of RBC-to-liquid separation with the ultrasonic standing wave. (**a**) Separation sequences including disaggregation, focusing, aggregation, and sedimentation. (**b**) Schematic of an ultrasonic transducer and square blood container (each width [*L*] = 12.3 mm). The ultrasonic transducer consists of a piezo resonator, carrier, and reflector. An ultrasonic standing wave of 2.1 MHz existed inside the blood container, and it remained perpendicular to the gravitational direction. (**C**) As a preliminary demonstration, the blood sample (normal RBCs in plasma, *Hct* = 50%, blood volume = 5 mL) was infused into the blood container. (**a**) Snapshots captured at a specific time (*t*) (*t* = 0, 300, 600, 900, and 1000 s) at *AP* = 0. (**b**) Snapshots captured at a specific time (*t*) (*t* = 0, 300, 600, 900, and 1000 s) at *AP* = 2 W. *Hs* represented the liquid height, and was used to quantify the RBC-to-liquid separation. (**c**) Temporal variations of *Hs* with respect to *AP* = 0 and 2 W.

As shown in Figure 1A, the experimental setup was composed of an ultrasonic system, a light illumination, and an image acquisition system. An ultrasonic system consisted of an ultrasonic transducer, and a controller (APS 990, AppliSens, Netherlands). The controller generated an electric signal with an excitation frequency of 2.1~2.15 MHz and transmitted it to the ultrasonic transducer. Right side panel showed piezo resonator in ultrasonic transducer. The acoustic power (*AP*) of the ultrasonic transducer was adjusted to *AP* = 0, 2, and 3 W by changing the voltage appropriately. A portable light-emitting diode (LED) light was employed to illuminate the RBCs present inside the ultrasonic transducer. A high-speed camera (FASTCAM MINI, Photron, Tokyo, Japan) was used to capture the cell-to-liquid separation (or separation height) inside the ultrasonic transducer. When a function generator (WF1944B, NF Corporation, Yokohama, Japan) triggered the high-speed camera at an interval of 1 s, the snapshot was captured sequentially at a frame rate of 250 Hz. The camera offered a spatial resolution of 1280 × 1000 pixels, and each pixel corresponded to 10 μm.

### 2.2. Design and Analysis of the Ultrasonic Transducer

As shown in Figure 1B(a), the PRF caused the disaggregated cells to rapidly move to either of the nodes or anti-nodes of pressure fields. It contributed to decreasing the average distance between the RBCs. The SRF was induced to aggregate the RBCs continuously until the gravity force of the aggregated RBCs overcame buoyancy and the sedimentation of RBCs began. In the transverse direction, the Bernoulli force (BF) played a significant role in packing the RBCs closely, as well as maintaining their positions. The aggregated RBCs tended to decrease [17]. The PRF in the axis direction (*F_r_*) was derived as follows:

(1) $$F_{r}=-\left(\frac{\pi p_{0}{}^{2}V_{c}\beta_{f}}{2\lambda }\right)\times \phi \left(\beta ,\;\rho \right)\times \sin\left(2kx\right)$$

and

(2) $$\phi \left(\beta ,\;\rho \right)=\frac{5\rho_{c}-2\rho_{f}}{2\rho_{c}+\rho_{f}}-\frac{\beta_{c}}{\beta_{f}}$$

In Equations (1) and (2), $p_{0}$, $V_{c}$, and *λ* represented the pressure amplitude, RBCs volume, and wavelength, respectively. The compressibility (*β*) was expressed as *β* = 1/(*ρ c*^2^). *ϕ* (*β, ρ*) referred to the acoustic contrast factor, which was determined by the density (*ρ_c_*, *ρ_f_*) and compressibility (*β_c_*, *β_f_*) of cells and fluid, respectively. A positive value of the acoustic contrast factor indicated that cells tended to move toward the node of pressure fields, whereas a negative value of the acoustic contrast factor indicated that the cell tended to move toward the anti-node of pressure fields. According to Equation (1), the PRF was proportional to $p_{0}$^2^ and $V_{c}$. 

As fluidic flow is considered as negligible in ultrasonic container, the motion of RBCs is determined by three forces (buoyant force, drag force, and gravity force) and size of the aggregated RBCs. Gravity force forces RBCs to move downward. However, as drag force and buoyant force act in upward direction, they retard RBCs sedimentation. When the gravity force of the aggregated RBCs overcomes buoyancy and the drag force, the sedimentation of RBCs begins. For the reason, sedimentation procedure of aggregated RBCs takes a longer time in an ultrasonic container.

A previous study reported that standing wave frequency with much lower than 1 MHz resulted in acoustic cavitation or shock waves. However, the pressure field with a standing wave frequency of 1~3 MHz did not impact the cell viability in mammalian cells and RBCs. For this reason, a standing wave frequency with *f* > 1 MHz was proposed in order to separate micron-sized cells effectively [18]. 

As shown in Figure 1B(b), the ultrasonic transducer consisted of a piezo resonator, carrier, blood container, and reflector. The blood container had a square shape (each length (*L*) = 12.3 mm). It was fabricated with transparent Pyrex material for visualizing RBC sedimentation. The resonator was bonded strongly to the carrier with an adhesive. Acoustic energy was transmitted sufficiently to the blood container because of the impedance matching functionality. The size and material property of the ultrasonic transducer are summarized as Table 1. 

In this study, the excitation frequency of the ultrasonic standing wave was kept constant at *f* = 2.1 MHz. When the blood container was filled with pure plasma or pure RBCs, the value of wavelength (*λ*) was in the range 0.75 ~ 0.78 mm. In addition, the acoustic contrast factor was calculated as a positive value (i.e., *ϕ* = 0.21 > 0). Because the wavelength was much greater than the size of RBCs, the acoustic standing wave led to acoustic streaming flow. Thus, the RBCs were focused and aggregated toward the nodes of acoustic pressure fields. 

### 2.3. RBC Separation and Sedimentation Using an Ultrasonic Transducer

As a preliminary demonstration, the blood sample (normal RBCs suspended in plasma, *Hct* = 50%, blood volume = 5 mL) was infused into the blood container. By setting the acoustic power as *AP* = 0 and 2 W, the RBCs-to-plasma separation was monitored over time. Figure 1C(a) shows snapshots captured at a specific time (*t* = 0, 300, 600, 900, and 1000 s) at *AP* = 0 (i.e., no ultrasonic standing wave). The experimental result indicates that the RBCs were not separated from the blood samples under a gravitational force. In other words, under the condition of no ultrasonic standing wave, the gravitational force alone did not contribute to the separation of RBCs from the blood sample (*Hct* = 50%). Figure 1C(b) shows the snapshots obtained at a specific time (*t* = 0, 300, 600, 900, and 1000 s) at *AP* = 2 W. The experimental result indicated that the acoustic radiation force contributed significantly to the separation of RBCs from the blood sample (*Hct* = 50%) when compared with the experimental results under pure gravitational force. To evaluate the RBC-to-plasma separation over time, the separation height (*Hs*) was defined as the liquid height. It was quantified with a digital image processing with Matlab (Ver. 2018a, Mathworks, USA). After a gray-scale image was converted into a binary-scale image using Otsu’s method [19], *Hs* was obtained by taking the arithmetic average of image intensity over the specific ROI (300 × 820 pixels) inside the blood container. As shown in Figure 1C(c), temporal variations of *Hs* were obtained with respect to *AP* = 0 and 2 W. Under no acoustic force (i.e., *AP* = 0), *Hs* was estimated as *Hs* = 1 mm after an elapse of 1100 s. However, when setting the acoustic power as *AP* = 2 W, *Hs* tended to increase significantly. The RBCs were separated largely from plasma (i.e., *Hs* = 7 mm at *t* = 1100 s). From the preliminary experiment, the acoustic radiation force contributed to the separation of RBCs or plasma from the blood sample sufficiently when compared with pure gravitational force.

## 3. Results and Discussion

### 3.1. Numerical Simulation for Estimating Acoustic Pressure Fields Inside Ultrasonic Transducer

**Figure 2:**
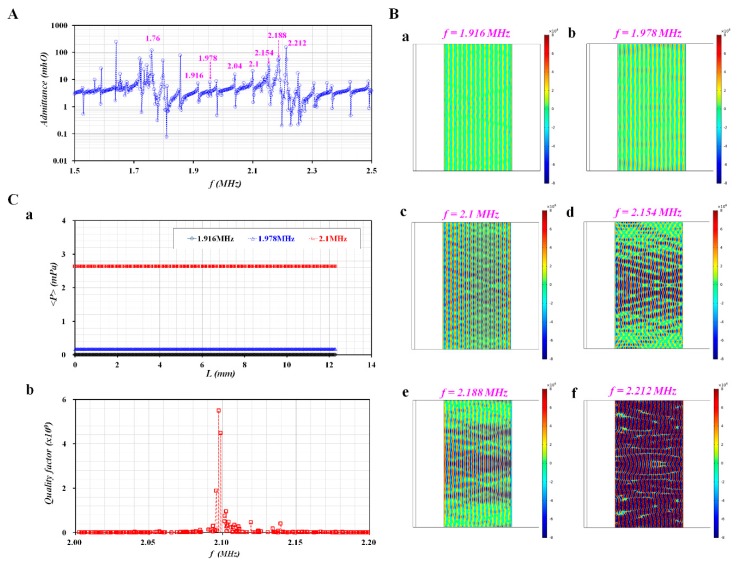
Numerical simulation of resonance frequency and acoustic pressure fields with respect to frequency. (**A**) Variations of admittance with respect to frequency for multilayer piezoelectric resonator. (**B**) Acoustic pressure fields at resonance frequency (*f*) ((**a**) *f* = 1.916 MHz, (**b**) *f* = 1.978 MHz, (**c**) *f* = 2.1 MHZ, (**d**) *f* = 2.154 MHz, (**e**) *f* = 2.188 MHz, and (**g**) *f* = 2.212 MHz). (**C**) Variations of averaged acoustic pressure (<*P*>) and quality factor with respect to excitation frequency values ranging from *f* = 2 MHz to *f* = 2.2 MHZ. (**a**) Variation of <*P*> with respect to *L* and *f* = 1.916, 1.978, and 2.1 MHz. (**b**) Variations of quality factor with respect to frequency.

When an AC potential was applied to the piezo resonator, the dynamic behavior of the ultrasonic transducer system was estimated as the electrical admittance with respect to frequency (*f*) [20]. Using the commercial software (COMSOL Ver. 5.4, COMSOL Inc., USA), variations in electric admittance were obtained by varying the frequency from *f* = 1.5 MHz to *f* = 2.5 MHz. Figure 2A shows the electrical admittance spectrum of the ultrasonic transducer (i.e., multi-layer resonator) filled with a blood sample. The spectrum represented that several resonant frequencies were present between *f* = 1.76 MHz and *f* = 2.212 MHz. The resonant frequency of 2.212 MHz was located near the fundamental resonant frequency of the piezo resonator (i.e., 2.2 MHz) [17]. According to the simulation results, overtone resonances with different amplitudes existed within fundamental frequencies of lead zirconate titanate PZT because the acoustic wavelength in the blood container was much smaller than the thickness of the reflector. Figure 2B shows variations in the acoustic pressure fields with respect to the resonant frequency ((a) *f* = 1.916 MHz, (b) *f* = 1.978 MHz, (c) *f* = 2.1 MHz, (d) *f* = 2.154 MHz, (e) *f* = 2.188 MHz, and (f) *f* = 2.212 MHz). This result indicated that three resonant frequencies (i.e., *f* = 1.916, 1.978, and 2.1 MHz) exhibited column mode in the perpendicular direction of acoustic wave propagation. Among them, a resonant frequency of 2.1 MHz had a higher amplitude of acoustic pressure fields. Thus, in this study, the excitation frequency of the controller was fixed at 2.1 MHz. The ultrasonic standing wave present in the blood container played a critical role in focusing and aggregating the RBCs at the node of acoustic pressure fields. Figure 2C(a) shows the averaged value of the acoustic pressure (<*P*>) for three resonant frequencies (i.e., *f* = 1.916, 1.978, and 2.1 MHz). As expected, the <*P*> exhibited a maximum value at *f* = 2.1 MHz. In addition, to induce a large displacement, it is required fundamentally to have a higher value of quality factor. Variations of quality factor were obtained by varying the frequency from 2 MHz to 2.2 MHz. As shown in Figure 2C(b), the simulation results indicated that the quality factor exhibited a maximum value at *f* = 2.098 MHz. From the numerical simulation, it was confirmed that *<P>* and quality factor had a maximum value near *f* = 2.1 MHz. Thus, the ultrasonic transducer showed maximum performance at an excitation frequency of 2.1 MHz, and yielded satisfactory performance in the range of resonant frequencies [21].

### 3.2. Experimental Visualization of RBCs Suspended in Ultrasonic Transducer

**Figure 3:**
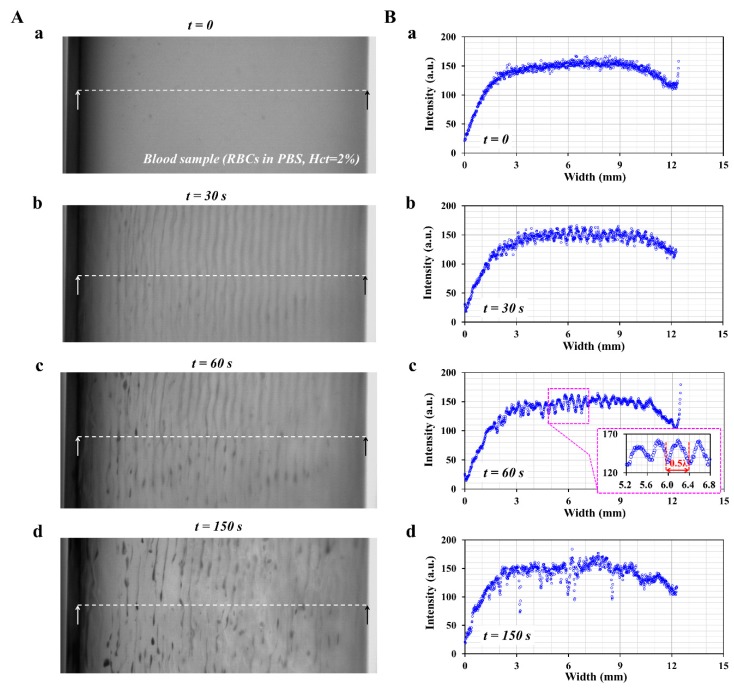
Experimental visualization of focusing and sedimentation of RBCs in ultrasonic transducer. (**A**) Snapshot captured at a specific time (*t*) ((**a**) *t* = 0, (**b**) *t* = 30 s, (**c**) *t* = 60 s, and (**d**) *t* = 150 s). (**B**) Variations of image intensity obtained along a straight line with respect to time (*t*) ((**a**) t = 0, (**b**) *t* = 30 s, (**c**) *t* = 60 s, and (**d**) *t* = 150 s). Inset of Figure 3B(c) shows periodic patterns of image intensity along the width direction.

To visualize the behaviors of RBCs suspended in the ultrasonic transducer, the blood sample was diluted extremely to *Hct* = 2% by adding normal RBCs to 1x PBS. The acoustic power of the controller was kept constant at *AP* = 2 W. Snapshots were then captured sequentially at intervals of 0.5 s. Figure 3A shows a snapshots captured at a specific time ((a) *t* = 0, (b) *t* = 30 s, (c) *t* = 60 s, and (d) *t* = 150 s). 

At an initial state (*t* = 0), there was no distinctive pattern with respect to RBCs. After *t* = 30 s, the RBCs were focused at several nodes of acoustic pressure fields. Several column modes are shown in the perpendicular direction of sound propagation. At *t* = 60 s, the RBCs were aggregated and formed clusters. After *t* =150 s, the RBC count tended to decrease significantly over time. To quantify the column mode of RBCs in the blood container, the image intensity was obtained along a straight line, as shown in each figure of Figure 3A. Figure 3B shows variations of the image intensity obtained along the width with respect to a specific time ((a) *t* = 0, (b) *t* = 30 s, (c) *t* = 60 s, and (d) *t* = 150 s). The RBC aggregation contributed to the varying image intensity values at each node. As shown in the inset of Figure 3B(c), the image intensity exhibited periodic patterns with respect to the width direction. This indicated that RBCs were aggregated at each node. To observe the variations in the image intensity, temporal variations in the image intensity were redrawn between 5.2 mm to 6.8 mm. As shown in the inset of Figure 3B(c), the periodic pattern was clearly illustrated to be along the direction of width. Subsequently, the half wavelength (i.e., 0.5 λ) was obtained as 0.5 λ = 0.378 ± 0.07 mm (N = 5). When compared with the half wavelength estimated from the acoustic model (i.e., 0.5 λ = 0.375 ~ 0.39 mm), there was little difference between the theoretical estimation and experimental result. Thus, it was estimated that the dynamic behavior of the ultrasonic transducer could be sufficiently estimated using the acoustic model.

### 3.3. Contributions of Acoustic Power to RBC-to-Liquid Separation

**Figure 4:**
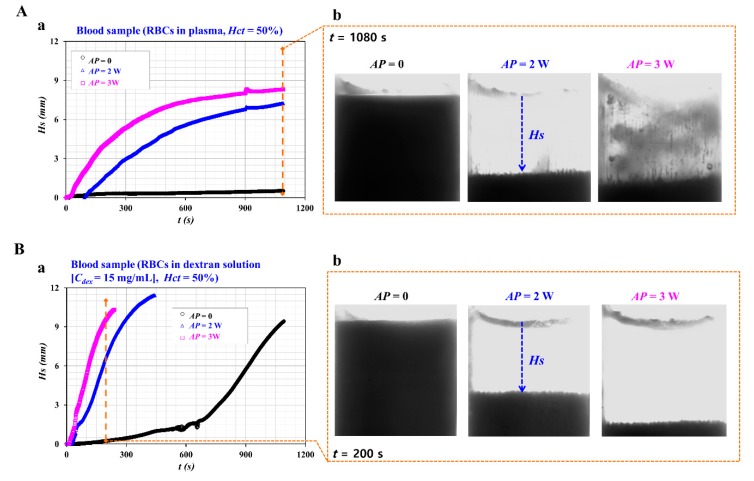
Contributions of acoustic power to RBC-to-liquid separation. (**A**) Variations of *Hs* for blood sample (Normal RBCs in plasma, *Hct* = 50%) with respect to the acoustic power (*AP*). (**a**) Temporal variations of *Hs* with respect to *AP*. (**b**) Snapshot captured at *t* = 1080 s with respect to *AP* r. (**B**) Variations of *Hs* for blood sample (Normal RBCs in dextran solution (*C_dex_* = 15 mg/mL), *Hct* = 50%) with respect to *AP*. (**a**) Temporal variations of *Hs* with respect to *AP*. (**b**) Snapshot captured at *t* = 200 s with respect to *AP*.

In the study, concentrated RBCs were purchased from the blood bank. The concentrated RBCs and 1x PBS were mixed and separated with centrifuge. Pure RBCs were then collected by removing buffy coat which included white blood cells or platelet. Then, blood sample was prepared by adding RBCs into various diluents (i.e., 1x PBS, plasma, and dextran solution). Thus, ultrasonic standing waves had an influence on RBCs inside ultrasonic transducer. It was necessary to evaluate the effect of the acoustic power on the RBC-to-liquid separation. A blood sample (*Hct* = 50%) was prepared by adding normal RBCs to the plasma, and blood sample was infused into the ultrasonic transducer. The acoustic power was set to *AP* = 0, 2, and 3 W. Figure 4A(a) shows temporal variations of *Hs* with respect to the acoustic power. In addition, Figure 4A(b) shows the snapshots captured at *t* = 1080 s with respect to the acoustic power. Under the condition of no acoustic power (i.e., *AP* = 0), no distinctive change in *Hs* was observed over time. However, when the acoustic power is set to *AP* = 2 W, *Hs* tended to increase substantially over time. Furthermore, when the acoustic power is from 2 W to 3 W, *Hs* achieved saturation rapidly. The results indicated that the acoustic power contributed to accelerating RBC-to-liquid separation significantly. When the RBCs were exposed to a higher value of acoustic power (i.e., *AP* = 3 W) for a longer duration of 1000 s, the heat generated by the piezo resonator contributed to adhering RBCs to the surface of the blood container. As shown in Figure 4A(b), the RBCs adhered to the surface of the blood container. The plasma was not shown transparently as a liquid layer. To mitigate this issue (i.e., thermal aggregation of RBCs), it was necessary to restrict the acoustic power below *AP* = 3 W, especially for a longer duration of operation (~1000 s). The power was therefore fixed at *AP* = 2 W, except when evaluating the effect of acoustic power on the separation efficiency. In addition, according to previous study [22], cavitation bubble was nucleated at low frequency of 200 kHz. Bubble size was estimated at 14 μm. However, at higher frequency with over 1 MHz, bubble size deceased to 1~3 μm significantly. However, throughout experiments, cavitation bubbles inside ultrasonic transducer was not shown clearly. There was no issue on RBCs damage resulting from bubble collapse.

As RBCs were suspended in a higher concentration of dextran solution (*C_dex_* = 15 mg/mL], the sedimentation rate of RBCs tended to increase significantly [23]. The blood sample was prepared once again by varying diluent from plasma to the dextran solution. Figure 4B(a) shows variations of *Hs* with respect to *AP* = 0, 2, and 3 W. Figure 4B(b) illustrates a snapshot captured at *t* = 200 s with respect to *AP*. Under no acoustic power, *Hs* tended to increase gradually because the dextran solution contributed to an increasing sedimentation rate of RBCs. When the acoustic power is set to *AP* = 2 or 3 W, *Hs* achieved saturation rapidly. From the results, the acoustic power contributed to increasing RBC-to-liquid separation significantly.

### 3.4. Contributions of Hematocrit and Dextran Solution to RBC-to-Liquid Separation

**Figure 5:**
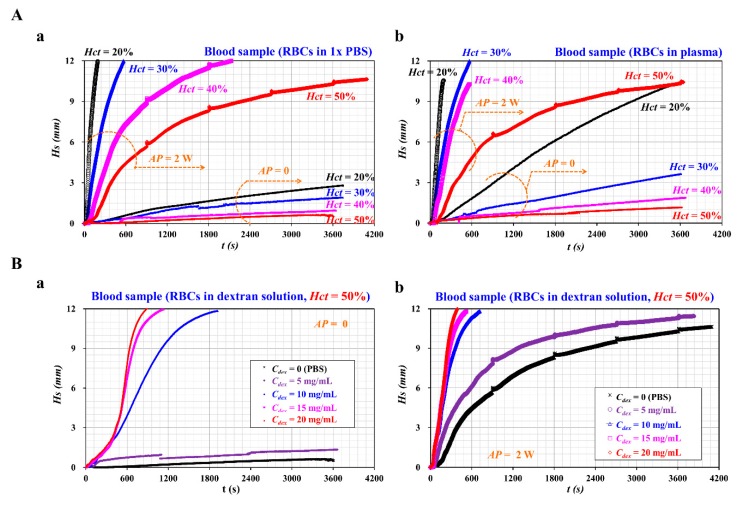
Effect of hematocrit and dextran solution on RBC-to-liquid separation. (**A**) Variations of *Hs* with respect to hematocrit (*Hct* = 20%, 30%, 40%, and 50%) and base solution (1x PBS, plasma). (**a**) Temporal variations of *Hs* of blood sample (normal RBCs in 1x PBS) with respect to acoustic power and hematocrit. (**b**) Temporal variations of *Hs* of blood sample (normal RBCs in plasma, *Hct* = 50%) with respect to acoustic power and hematocrit. (**B**) Variations of *Hs* with respect to concentration of dextran solution (*C_dex_* = 0, 5, 10, 15, and 20 mg/mL) and acoustic power (0, 2 W). (**a**) Temporal variations of *Hs* with respect to *C_dex_* and acoustic power = 0. (**b**) Temporal variations of *Hs* with respect to *C_dex_* and acoustic power = 2 W.

First, as hematocrit hindered the sedimentation rate of RBCs in the stationary flow condition, it was necessary to evaluate the effect of hematocrit on the RBC-to-liquid separation under acoustic power condition. A blood sample with *Hct* = 20%, 30%, 40%, and 50% was prepared by adding normal RBCs to diluent (i.e., 1x PBS, and plasma). To eliminate the effect of plasma protein on the sedimentation rate of RBCs, a blood sample was prepared by adding normal RBCs to 1x PBS. Figure 5A(a) shows temporal variations of *Hs* with respect to the acoustic power and hematocrit. Under no acoustic power, *Hs* changed slightly over time. Variations of *Hs* tended to decrease at higher values of hematocrit. In other words, a blood sample with *Hct* = 20% exhibited approximately *Hs* = 3 mm after an elapse of 3600 s. Furthermore, a blood sample with *Hct* = 50% exhibited an *Hs* value of approximately 1 mm after a duration of 3600 s. However, under an acoustic power of *AP* = 2 W, *Hs* achieved a saturation height (i.e., *Hs* = 12 mm) within a short time. The saturation time (*T_S_*) of the corresponding hematocrit was estimated to be *T_S_* = 200 s (*Hct* = 20%), *T_S_* = 600 s (*Hct* = 30%), and *T_S_* = 2100 s (*Hct* = 40%). For the blood sample with *Hct* = 50%, *Hs* tended to increase greatly. However, it did not achieve the saturation height after an elapse of 4100 s. The results indicated that the acoustic power was considered as effective for separating the RBCs from the blood sample. The value of *Hs* was therefore inversely proportional to the hematocrit level. According to a previous study [21], when RBCs were suspended into plasma rather than 1x PBS, the sedimentation rate of RBCs increased substantially. To verify the variation of the sedimentation rate, the blood sample was prepared by adding normal RBCs to the plasma. Figure 5A(b) shows temporal variations of *Hs* with respect to the acoustic power and hematocrit. When compared with the results shown in Figure 5A(a), plasma contributed to increasing *Hs* significantly. The saturation time tended to decrease significantly with respect to *Hct* = 20%, 30%, and 40%. Interestingly, the blood sample (*Hct* = 50%) did not show a significant difference depending on diluent. As the higher value of hematocrit hindered the sedimentation rate of RBCs significantly, plasma did not have a strong influence on variations of *Hs* when compared with 1x PBS. Based on the results, it was found that variations of *Hs* tended to decrease at a higher level of hematocrit. The acoustic power played a significant role in accelerating *Hs* over time. Under an acoustic power of 2 W, when compared with 1x PBS, plasma contributed to increasing variations of *Hs*, especially below *Hct* = 50%. 

Second, to evaluate the effect of acoustic power on the blood sample (*Hct* = 50%), dextran solution was employed to increase the sedimentation rate of RBCs instead of 1x PBS or plasma. To significantly enhance the sedimentation of RBCs in the blood sample (*Hct* = 50%), various concentrations of dextran solution (*C_dex_* = 0, 5, 10, 15, and 20 mg/mL) were prepared by adding dextran powder to 1x PBS. Here, *C_dex_* = 0 meant 1x PBS. Hematocrit of blood sample was adjusted to 50% by adding normal RBCs into each dextran solution. Figure 5B(a) shows temporal variations of *Hs* with respect to *C_dex_* under no acoustic power (*AP* = 0). Above *C_dex_* = 10 mg/mL, *Hs* increased significantly over time. It achieved saturation height rapidly at a higher concentration of dextran solution. The blood sample (*C_dex_* = 5 mg/mL) contributed to a slight increase in *Hs* when compared with the blood sample (1x PBS). Under an acoustic power of 2 W, variations of *Hs* were obtained to evaluate the contributions of the acoustic power with respect to *C_dex_*. Figure 5B(b) represents temporal variations of *Hs* with respect to *C_dex_* under *AP* = 2 W. As a result, the acoustic power contributed to increasing *Hs* substantially over time. Thus, with respect to dextran solution of *C_dex_* = 10, 15, and 20 mg/mL, *Hs* arrived at a saturation height within 700 s. In addition, a lower concentration of dextran solution (*C_dex_* = 5 mg/mL) contributed to a slight increase in *Hs* when compared with the control blood sample (1x PBS). Based on the result, dextran solution contributed to increasing RBC-to-liquid separation significantly, even for a blood sample with *Hct* = 50%. An acoustic power of 2 W played a significant role in accelerating the sedimentation of RBCs substantially when compared to the case with no acoustic power.

### 3.5. Hardened Blood Sample Preparation with Ultrasonic Transducer

**Figure A2:**
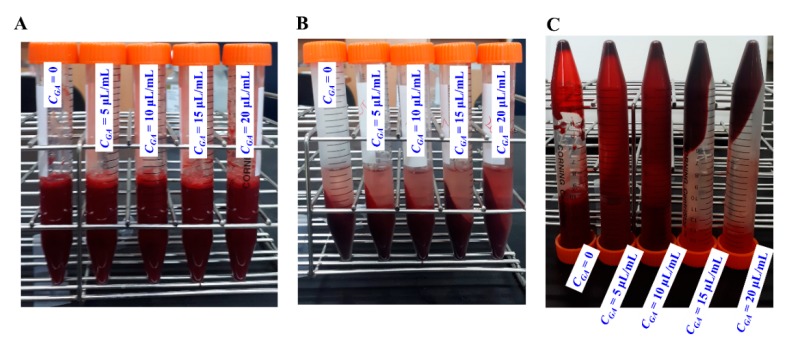
**RBCs stacked and adhered to surface of tube after operating centrifuge.** Blood sample (*Hct* = 50%) was prepared by adding normal RBCs into a specific concentration of GA solution (*C_GA_* = 0, 5, 10, 15, and 20 μL/mL). (**A**) Tubes filled with blood samples before operating centrifuge. (**B**) Tubes filled with blood samples after operating centrifuge (4000 rpm x 10 min). (**C**) Inverted tubes filled with hardened blood samples with respect to C_GA_. Above *C_GA_* =15 μL/mL, hardened RBCs adhered to the surface of tubes, and it was difficult to collect hardened RBCs from the tube.

When preparing fixed RBCs with glutaraldehyde (GA) solution, normal RBCs were added and mixed to a specific concentration of GA solution. After an elapse of specific time, normal RBCs were fixed. To collect fixed RBCs, a centrifugal separator was usually employed to separate fixed RBCs and liquid from the blood sample. During the centrifugal operation, RBCs were stacked and adhered to the surface of the tube because of the strong centrifugal forces in outward direction. Thus, it was difficult to collect fixed RBCs from the tube. As shown in Figure A2A (Appendix A), the blood sample (*Hct* = 50%) was prepared by adding normal RBCs to a specific concentration of GA solution (*C_GA_* = 0, 5, 10, 15, and 20 μL/mL). After fitting tubes into a centrifuge, it rotated at 4000 rpm for 10 min. Figure A2B (Appendix A) shows tubes filled with blood samples after operating the centrifuge. Owing to the centrifugal force, RBCs were positioned diagonally inside a tube. To evaluate the RBC adhesion simply, a tube was inverted at about 180°. As shown in Figure A2C (Appendix A), the blood sample (i.e., fixed RBCs with *C_GA_* <= 10 μL/mL) flowed downward owing to the gravitational force. The fixed RBCs were then collected after removing liquid as the upper layer. However, the blood sample (i.e., fixed RBCs with *C_GA_* values above 15 μL/mL) did not flow downward because RBCs adhered to the inner surfaces of the tube. For the reason, it was difficult to collect fixed RBCs from the tube. 

**Figure 6:**
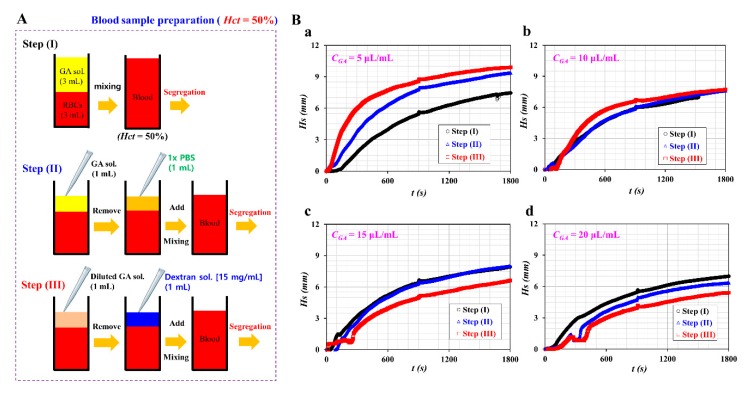
Measurement of separation height (*Hs*) after adding normal RBCs into GA solution, 1x PBS, and dextran solution (*C_dex_* = 15 mg/mL). (**A**) Schematic of the three steps for preparing blood sample. (**a**) Step (I): normal RBCs (3 mL) and a specific concentration of GA solution (3 mL) were infused and mixed in blood container. (**b**) Step (II): after operating ultrasonic transducer for 30 min, GA solution (~1 mL) was removed from top layer. 1x PBS was then added and mixed in blood container. (**c**) Step (III): after operating ultrasonic transducer for 30 min, diluted GA solution (~1 mL) was removed from the top layer. A specific concentration of dextran solution (*C_dex_* = 15 mg/mL) was added and mixed in blood container. (**B**) Temporal variations of *Hs* for the blood sample (*C_GA_* = 5 μL/mL) following the sample preparation process [step (I), step (II), and step (III)] for the blood sample mixed with normal RBCs and *C_GA_*: (**a**) C_GA_ = 5 μL/mL, (**b**) C_GA_ = 10 μL/mL, (**c**) C_GA_ = 15 μL/mL, and (**d**) C_GA_ = 20 μL/mL.

When compared with the centrifuge, the ultrasonic transducer did not apply a strong force to the RBCs. In other words, as the ultrasonic radiation force was much smaller than the centrifugal force, the contributions of ultrasonic radiation force to the RBC adhesion were sufficiently negligible. Inside the blood container, RBCs come down inversely in gravitational direction and stacked with the ultrasonic transducer. Using the fixed blood sample discussed in Figure A2 (Appendix A), the RBC-to-liquid separation was evaluated using the ultrasonic transducer. As shown in Figure 6A, blood sample preparation was divided into three stages: step (I), step (II), and step (III). Throughout the three stages, the diluent was changed only after operating the ultrasonic transducer. The hematocrit of the blood sample was fixed at *Hct* = 50%. First, Figure 6A(a) illustrates the procedure involved in the blood sample preparation in step (I). Normal RBCs (~3 mL) and a specific concentration of GA solution (~ 3 mL) were added and mixed in a blood container. To sufficiently fix the membrane of normal RBCs, blood sample had been remained for 10 min. After operating the ultrasonic transducer for 30 min, the blood sample was separated into RBCs (in the lower layer) and the GA solution (in the upper layer). Second, Figure 6A(b) shows the procedure of blood sample preparation in step (II). The GA solution (~1 mL) was removed from the upper layer. 1x PBS (~ 1 mL) was then added and mixed in a blood container. After operating the ultrasonic transducer for 30 min, the blood sample was separated into RBCs and GA solution diluted with 1x PBS. Third, Figure 6A(c) shows the procedure of blood sample preparation at step (III). The diluted GA solution (~1 mL) was removed from the top layer. A specific concentration of dextran solution (*C_dex_* = 15 mg/mL) (~ 1 mL) was then added and mixed in the blood container. After operating the ultrasonic transducer for 30 min, the blood sample was separated into RBCs and liquid. 

With respect to the three stages, the temporal variations of *Hs* for the blood sample were obtained by varying the concentration of the GA solution. Figure 6B(a) shows the temporal variations of *Hs* with respect to each step. Here, at step (I), the GA solution of 5 μL/mL was used to fix the membrane of normal RBCs. The result indicated that *Hs* tended to increase gradually by changing the diluent (i.e., GA solution → 1x PBS → dextran solution). Figure 6B(b) shows temporal variations of *Hs* for the hardened blood sample with C_GA_=10 μL/mL. Step (I) and step (II) did not show substantial differences with respect to the variation of *Hs* over time. Compared with step (I) and step (II), step (III) (i.e., dextran solution as diluent) exhibited a slight increase of *Hs* over time. However, as shown in Figure 6B(c,d), with respect to the higher concentration of GA solution (*C_GA_* = 15, and 20 μL/mL), step (III) shows a decrease of *Hs* over time. According to the experimental results, *Hs* tended to decrease gradually at higher concentrations of the GA solution. Below C_GA_ = 10 μL/mL, the dextran solution [step (III)] contributed to an increasing *Hs*. However, above *C_GA_* = 15 μL/mL, the dextran solution contributed to a gradual decrease in *Hs*. At a higher concentration of GA solution, fixed RBCs did not contribute to RBC aggregation. In other words, the dextran solution did not contribute to the acceleration of *Hs* over time. During the procedure of blood sample preparation, dextran solution was added to dilute GA solution at step (III). The suctioning or discharging of the blood sample with a pipette required a large force. For the reason, it was inferred that the mixed solution between GA solution and dextran solution may hinder significantly the RBC-to-liquid separation.

From the experimental results, the ultrasonic transducer could be used effectively to separate the hardened blood sample without RBC adhesion or the occurrence of stacks while operating the centrifuge.

### 3.6. Measurement of RBC Aggregation and Blood Viscosity for Hardened Blood Sample 

As discussed with respect to Figure 6, a blood sample was collected at each step. For each step, the blood sample was collected as fixed RBCs suspended in GA solution (step [I]), fixed RBCs suspended in 1x PBS (step [II]), and fixed RBCs suspended in dextran solution (step [III]). In other words, RBCs and diluent were separated by operating ultrasonic transducer. Blood sample was prepared by adding RBCs into specific diluents (i.e., GA solution, 1x PBS, and dextran solution). Blood sample and 1x PBS were infused simultaneously by controlling flow rate of both fluids with syringe pumps. While varying diluent at each step, RBC aggregation and blood viscosity were measured simultaneously. 

**Figure 7:**
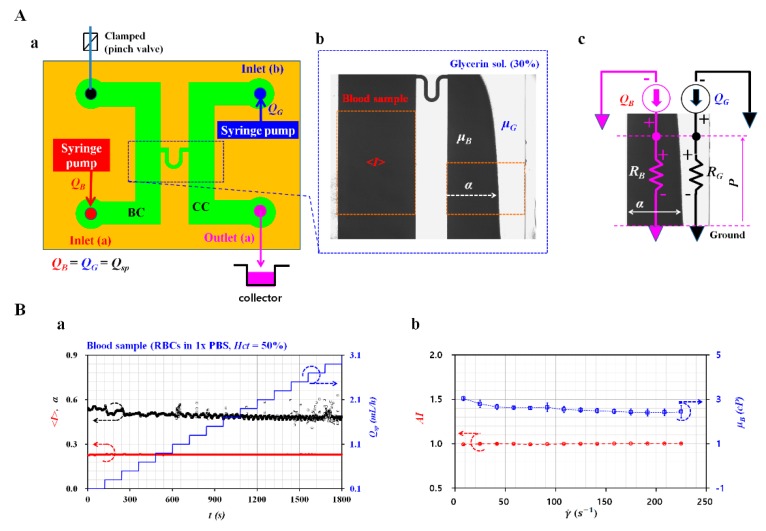
Quantification of RBC aggregation **(***AI)* and blood viscosity (*μ_B_*) in microfluidic environment. (**A**) Simultaneous measurement of *AI* and *μ_B_* with microfluidic device. (**a**) Schematic of experimental setup for measuring *AI* and *μ_B_*. (**b**) Quantification of microscopic image intensity (*<I>*) in blood channel and interface (*α*) in coflowing channel. (**c**) Discrete fluidic circuit model for blood viscosity in coflowing channel. (**B**) Quantification of *AI* and *μ_B_* for normal blood sample (normal RBCs in 1x PBS, Hct = 50%). (**a**) Variations of *<I>*, *α*, and Q_sp_ with an elapse of time. (**b**) Variations of *AI* and *μ_B_* over shear rate.

Figure 7A(a) shows the experimental setup for measuring RBC aggregation and blood viscosity. The experimental setup was composed of microfluidic device, and two syringe pumps. As shown in Figure 7A(b), the image intensity (*<I>*) in the blood channel (BC) was obtained to monitor RBC aggregation with respect to the blood flow rate. In addition, the blood viscosity in the coflowing channel (CC) was obtained by evaluating the interface. The blood sample (i.e., test fluid) and 30% glycerin solution (i.e., reference fluid) were injected by controlling two syringe pumps at the same flow rate (i.e., *Q_B_* = *Q_G_* = *Q_SP_*, *Q_B_*: flow rate of blood sample, *Q_G_*: flow rate of glycerin solution, and *Q_SP_*: flow rate of syringe pump). The flow rate of syringe pump increased gradually from *Q_SP_* = 0.1 mL/h to *Q_SP_* = 3.1 mL/h at an interval of 0.2 mL/h [24]. Hematocrit of blood sample was adjusted to 50% by adding normal RBCs to 1x PBS. As shown in Figure 7A(c), a simple mathematical model was constructed with discrete fluidic circuit elements (i.e., flow rate (*Q_B_*, *Q_G_*), and resistance element (*R_B_*, *R_G_*). Based on the sample pressure of both fluids in the coflowing channel, the blood viscosity (*μ_B_*) was expressed as $\mu_{B}=\mu_{G}\times CF\left(\alpha \right)\times \frac{\alpha }{1-\alpha }$. Here, correction factor (*CF*) was included to compensate the boundary difference between real physical model and simple mathematical model. As shown in Figure 7B(a), variations of *<I>*, *<α>*, and *Q_sp_* were obtained with respect to time. As 1x PBS did not include plasma protein, *<I>* remained constant under a varying flow rate. *<α>* tended to decrease at a higher flow rate. According to a previous study [24], *AI* and *μ_B_* were obtained from temporal variations of *<I>* and *<α>* respectively. Figure 7B(b) shows variations of AI and *μ_B_* with respect to shear rate ($\dot{\gamma }$). As expected, AI remained constant with respect to $\dot{\gamma }$. In addition, *μ_B_* tended to decrease gradually with respect to $\dot{\gamma }$ because the blood sample behaved as a non-Newtonian fluid. 

**Figure 8:**
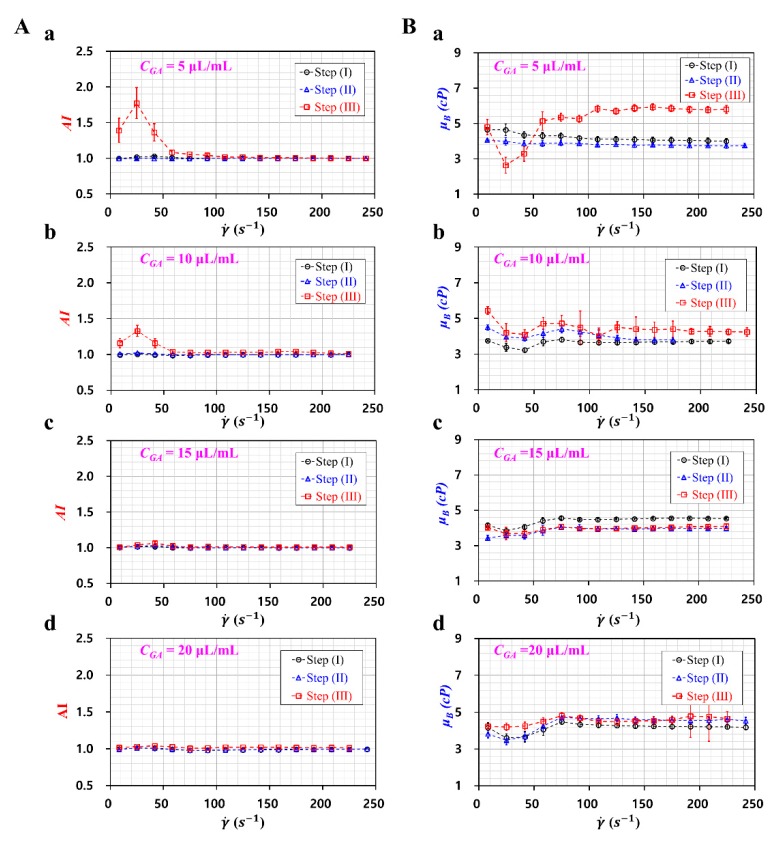
Quantitative evaluations of *AI* and blood viscosity for fixed blood sample with different diluents with respect to step (I, II, and III). (**A**) Variations of AI with respect to *C_GA_* ((**a**) *C_GA_* = 5 μL/mL, (**b**) *C_GA_* = 10 μL/mL, (**c**) *C_GA_* = 15 μL/mL, and (**d**) *C_GA_* = 20 μL/mL) and shear rate. (**B**) Variations of *μ_B_* with respect to *C_GA_* ((**a**) *C_GA_* = 5 μL/mL, (**b**) *C_GA_* = 10 μL/mL, (**c**) *C_GA_* = 15 μL/mL, and (**d**) *C_GA_* = 20 μL/mL) and shear rate.

First, to evaluate the effects of RBCs or diluent on RBC aggregation, RBC aggregation were obtained by changing diluents (i.e., step (I), step (II), and step (III)) and GA solution (i.e., *C_GA_* = 5, 10, 15, and 20 μL/mL). As shown in Figure 8A, step (I) and step (II) did not exhibit variations of AI with respect to shear rate. However, step (III) shows that AI tended to decrease largely for up to *C_GA_* =10 μL/mL. Above *C_GA_* = 15 μL/mL, there was little variation of *AI* with respect to *C_GA_*. From this result, fixed RBCs with GA solution of *C_GA_* = 15~20 μL/mL did not contribute to the RBC aggregation. 

Second, to evaluate the contribution of RBCs or diluent to the blood viscosity, variations in the blood viscosity were obtained by varying the diluent (i.e., step (I), step (II), and step (III)) and *C_GA_*. As shown in Figure 8B, variations of *μ_B_* were obtained with respect to *C_GA_* and shear rate. At lower shear rates (i.e., $\dot{\gamma }$ < 100 $s^{-1})$, *μ_B_* showed large variations with respect to shear rate. RBC aggregation posed difficulties when evaluating the interface between the blood sample and 30% glycerin solution. Thus, it led to reduced measurement accuracy. However, at a higher shear rate, *μ_B_* showed smaller variations over the shear rate. As shown in Figure 8B(a), step (III) shows an increase of *μ_B_* when compared with step (I) and step (II). As the syringe pump was installed vertically, the erythrocyte sedimentation rate (ESR) occurred in the driving syringe. Because of the continuous ESR in the driving syringe, the hematocrit of the blood sample infused in the microfluidic channel increased over time. In other words, dextran solution contributed to increasing RBC aggregation, which resulted in an increase in the hematocrit and blood viscosity. As shown in Figure 8B(b), the dextran solution resulted in an increased blood viscosity. However, the variation of the blood viscosity was relatively reduced when compared with Figure 8B(a). This result indicated that the effect of dextran solution on blood viscosity tended to decrease at higher concentrations of GA solution (i.e., fixed RBCs). As shown in Figure 8B(c,d), dextran solution did not contribute to the increase in blood viscosity. In addition, the blood viscosity remained constant over the shear rate. In other words, as fixed RBCs did not include flexibility with respect to the shear rate, the blood viscosity remained constant over the shear rate.

From the aforementioned experimental results, it lead to the conclusion that RBC aggregation and blood viscosity measured by the microfluidic technique could be employed to monitor fixed blood samples. In addition, the dextran solution contributes to increasing RBC aggregation and blood viscosity below certain concentrations of GA solution (i.e., *C_GA_* = 5 and 10 μL/mL). 

## 4. Conclusions

In this study, to separate RBCs and liquid from the blood sample, an ultrasonic transducer was proposed instead of a centrifuge. Using the microfluidic technique, RBC aggregation and blood viscosity were obtained with respect to each blood sample. First, a numerical simulation was conducted to evaluate the characteristics of the ultrasonic transducer (i.e., natural frequency, acoustic pressure fields, and quality factor). Based on the variations in the acoustic power (*<P>*) and quality factor with respect to frequency, the ultrasonic transducer showed a maximum performance at an excitation frequency of 2.1 MHz. Second, the periodic pattern was visualized as a column mode experimentally. The half wavelength was found to be 0.5 λ = 0.378 ± 0.07 mm. The experimental result was in good agreement with the analytical estimation. Third, the acoustic power effectively separated the RBCs or base solution from various blood samples (i.e., *Hct* = 20% ~ 50%, base solution: plasma, 1x PBS, and dextran solution). The experimental results indicated that RBCs with hematocrit of 50% were separated substantially within 10 min under acoustic power of 2 W. Additionally, ESR (erythrocyte sedimentation rate) was highly accelerated under acoustic pressure fields. Fourth, the present method was employed to separate fixed RBCs from fixed blood samples composed of fixed RBCs suspended in GA solution. According to experimental results, the ultrasonic transducer did not induce RBC adhesion or the occurrence of stacks. The present method separated fixed RBCs from fixed blood sample easily. Instead of the centrifuge, the proposed method was employed to prepare various fixed blood samples while changing diluents (i.e., GA solution, 1x PBS, and dextran solution). Then, RBC aggregation and the blood viscosity of the blood sample were obtained successfully by varying the blood samples. In conclusion, the present method showed promising potentials for blood sample preparation with ultrasonic-based separation, and quantification of mechanical properties of blood samples in microfluidic device. In the near future, if a microfluidic platform and ultrasonic transducer will be integrated into a closed fluidic circuit, multiple mechanical properties of blood samples could be monitored simultaneously for a certain longer period. 

## Figures and Tables

**Table 1:** Summary of size and material property of an ultrasonic transducer.

Layer	Piezoelectric	Carrier	Blood Container		Reflector
ℓ (mm)	0.5	5.14	12.3		5.14
Material	PIC181	Pyrex	Plasma	RBCs	Pyrex
ρ (kg/m^3^)	7850	2200	1025	1093	2200
v (m/s)	4460	5400	1570	1645	5400
Remark	As acoustic contrast factor took positive value (ϕ = 0.21), RBCs tended to focus and aggregate at the nodes of pressure fields.				

**Figure A1:**
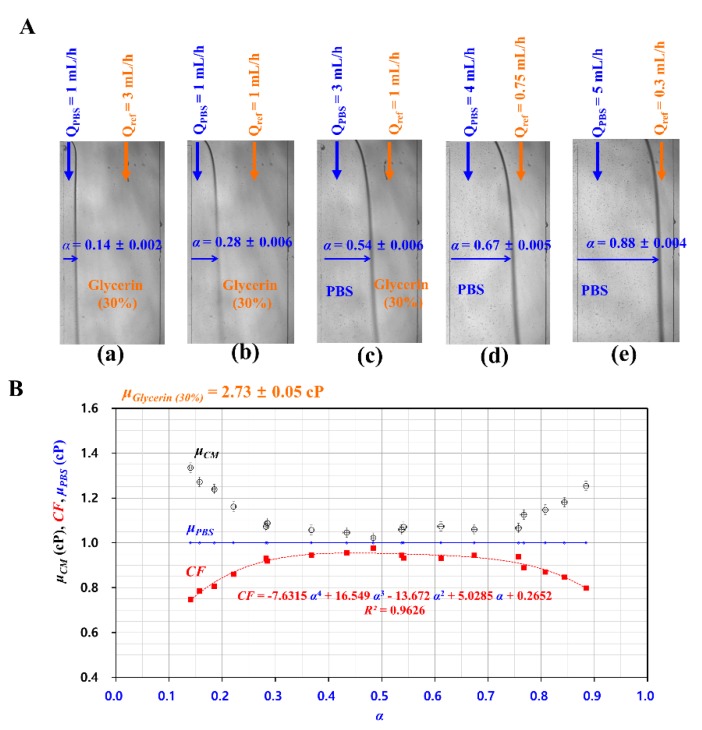
Correction factor of parallel flow method estimated by conducting experimental results. Here, a flow rate of 1x PBS (test fluid, *Q_PBS_*) remained constant with a syringe pump. Simultaneously, the flow rate of 30% glycerin solution (reference fluid, *Q_ref_*) remained constant with a syringe pump. The interface (*α*) in coflowing streams in a coflowing channel was monitored by analyzing microscopic images captured over time. (**A**) Variations of *α* with respect to the flow rate ratio (*Q_PBS_*/*Q_ref_*). The *α* value of the corresponding flow rate ratio was obtained as (**a**) *α* = 0.14 ± 0.002 (*Q_PBS_/Q_ref_* = 1/3), (**b**) *α* = 0.28 ± 0.006 (*Q_PBS_/Q_ref_* = 1/1), **(c)** *α* = 0.54 ± 0.006 (*Q_PBS_/Q_ref_* = 3/1), (**d**) *α* = 0.67 ± 0.005 (*Q_PBS_/Q_ref_* = 4/0.75), and (**e**) α = 0.88 ± 0.004 (*Q_PBS_/Q_ref_* = 5/0.3). (**B**) Variations of the correction factor (*CF*) with respect to *α*. A viscosity of 30% glycerin solution was given as *μ_glycerin (30%)_* = 2.73 ± 0.05 cP. According to the coflowing method without the correction factor, the analytical expression of *μ_CM_* was given as $\mu_{CM}=\mu_{Glycerin\;\left(30\%\right)}\times \frac{\alpha }{1-\alpha }$ The *μ_CM_* results showed large deviations of 30% at both walls. To obtain the viscosity of 1x PBS as *μ_PBS_* = 1 cP along the interface ranging from *α* = 0.14 to *α* = 0.88, correction factor (*CF*) was found to be *CF* = 1/*μ_CM_*. According to the regression analysis, the polynomial expression of *CF* was determined to be *CF* = -7.6315 *α^4^* + 16.549 α^3^ - 13.672 *α^2^* + 5.0285 α + 0.2652 (*R^2^* = 0.9626).

## Appendix A

### Appendix A.1. Blood Sample Preparation

According to Ethics Committee of Chosun University Hospital (CHOSUN 2018-05-11), it was confirmed that all experimental procedures were appropriate and humane.

Concentrated RBCs and fresh frozen plasma (FFP) were provided from the Gwangju–Chonnam blood bank (Gwangju, South Korea). Because the concentrated RBCs consisted of white blood cells, platelets, and citrate phosphate dextrose adenine (CPDA), several washings were conducted to prepare pure RBCs by removing the other cells. The RBCs (~7 mL) and 1x PBS (~7 mL) were agitated sufficiently in a 15-mL tube. After the tube was fixed into a centrifuge (Allegra X-30R benchtop, Beckman Coulter, Brea, CA, USA), it was rotated at 4000 rpm for 10 min. Pure RBCs (i.e., the lower layers) were then collected after removing the buffy layer (white blood cells, and platelet) and 1x PBS. To adequately remove the CPDA, the washing procedure was repeated twice. Because FFP was kept at −20 °C, it was thawed to a room temperature of 25 °C. After filtering the debris or larger cells with a syringe filter (filter mesh size = 5 μm), plasma was then collected in a 15-mL tube. The RBCs and plasma were stored at 4 °C prior to conducting the blood test.

To evaluate the performance of the ultrasonic transducer, various blood samples were prepared carefully by changing the base solution (i.e., 1x PBS, plasma, and the specific concentration of dextran solution) and RBCs (i.e., normal RBCs or RBCs with different degrees of hardness). First, to measure the contributions of hematocrit to the cell-to-liquid separation (i.e., separation efficiency), the hematocrit of the blood sample was adjusted to *Hct* = 30%, 40%, and 50% by adding normal RBCs to the base solution (1x PBS, or plasma). Second, to enhance the degree of RBC aggregation, four concentrations of dextran solution ranging from 5 mg/mL to 20 mg/mL at intervals of 5 mg/mL were prepared by adding dextran powder (*Leuconostoc* spp., MW = 450–650 kDa, Sigma–Aldrich, USA) to 1× PBS. Blood sample with *Hct* = 50% was prepared by adding normal RBCs to each dextran solution. Third, to decrease RBCs deformability, normal RBCs were fixed with a glutaraldehyde (GA) solution. Four concentrations of GA solution ranging from 5 µL/mL to 20 µL/mL at intervals of 5 µL/mL were diluted by adding GA solution (Grade II, 25% in H_2_O, Sigma-Aldrich, USA) to 1x PBS. Subsequently, for cell-to-liquid separation step, the blood sample was prepared by adding fixed RBCs to diluents (i.e., 1x PBS, and dextran solution of 15 mg/mL).

### Appendix A.2. Fabrication and Procedures for Microfluidic-Based Experiments

To measure RBC aggregation and blood viscosity under a microfluidic platform, a microfluidic device consisted of two channels (blood channel, and coflowing channel), and two inlets (a, b), one process inlet (a), and one outlet (b). As shown in Figure 7A(a), the blood channel (width = 2 mm, length = 22.5 mm) was connected to the coflowing channel (width = 2 mm, length = 22.5 mm) through a bridge channel (width = 100 μm, length = 1750 μm) [25]. The depth of this channel was fixed at 100 μm.

A four-inch silicon mold was prepared with micro-electromechanical-system techniques, including photolithography and deep reactive ion etching. PDMS (polydimethylsiloxane) (Sylgard 184, Dow Corning, Midland, MI, and USA) elastomer and curing agent were mixed at a ratio of 10:1. Air bubbles dissolved in the PDMS mixture were removed with a vacuum pump for 1 h. After curing the PDMS mixture in a convective oven (70 °C, and 1 h), a cured PDMS block was peeled off from the mold and cut with a razor blade. Two inlets (a, b), one process (a), and one outlet (a) were then punched with a biopsy punch (outer diameter = 1.0 mm). Using an oxygen plasma system (CUTE-MPR, Femto Science Co., South Korea), the PDMS block was bonded to the glass slide.

A polyethylene tubing (inner diameter = 500 μm, and length = 300 mm) were inserted tightly to individual inlet (a, b). The other polyethylene tubing (inner diameter = 500 μm, and length = 200 mm) was fitted tightly to outlet (a). The last polyethylene tube (inner diameter = 500 μm, and length = 10 mm) was fitted tightly to a process inlet (a). The channel was filled with 2 mg/mL bovine serum albumin solution through a process inlet (a). After an elapse of five minutes, microfluidic channels were again filled with 1× PBS. The other end of the tube connected to the process inlet (a) was clamped with a pinch valve. Two syringes (~1 mL) were filled with blood sample (i.e., test fluid) and glycerin solution (i.e., reference fluid). Two syringes were installed vertically with respect to the gravitational direction. After installing two syringes into the corresponding syringe pump (neMESYS, Cetoni GmbH, Germany), the blood sample and 30% glycerin solution were infused at the same flow rate (i.e., *Q_B_* = *Q_G_* = *Q_SP_*). The flow rate increased stepwise from *Q_SP_* = 0.1 mL/h to *Q_SP_* = 3.1 mL/h at an interval of 0.2 mL/h. Each flow rate was kept constant for 2 min [8,24].

A microfluidic device was subsequently positioned on an optical inverted microscope (BX51, Olympus, Tokyo, Japan) equipped with a 4× objective lens (NA = 0.1). A high-speed camera was employed to capture the microscopic image intensity of the blood sample flowing in the blood channel, and to monitor the interface between the blood sample and 1x PBS in the coflowing channel. The microscopic images were captured at a frame rate of 500 Hz. A function generator triggered the high-speed camera at intervals of 1 s. Two microscopic images were captured sequentially at intervals of 1 s. All experiments were conducted at a temperature of 25 °C.

### Appendix A.3. Quantification of RBC Aggregation and Blood Viscosity in Microfluidic Channels

First, to monitor variations of the RBC aggregation, the image intensity of the blood sample flowing in the BC was obtained by quantifying the microscopic images of RBCs distributed in a specific region-of-interest (ROI) with 483 x 700 pixels, as shown in Figure 7A(b). By conducting digital image processing with Matlab (Ver. 2018a, Mathworks, USA), the averaged image intensity (<*I*>) was obtained by conducting the arithmetic average of the image intensity over the specific ROI in blood channel. As RBCs were disaggregated fully at a higher shear rate, the <*I*> tended to decrease at a higher shear rate. After obtaining the minimum value of the image intensity *<I>_min_* at a higher shear rate (i.e., $\dot{\gamma }>100\;s^{-1})$, the RBC aggregation index (*AI*) was obtained by dividing the image intensity obtained at each flow rate (<*I*>) by *<I>_min_* (i.e., *AI* = *<I>* / *<I>_min_*) [24].

Second, to evaluate the variations in the blood viscosity, a specific ROI with 483 x 250 pixels was selected in the coflowing channel, as shown in Figure 7A(b). After a gray-scale image was converted into a binary-scale image with Otsu’s method [16], the averaged value of the interface in coflowing channel (<*α*>) was calculated by averaging *α* distributed in the ROI. As shown in Figure 7A(c), two fluids flowing in coflowing channel was modeled with discrete fluidic circuit model. Both fluids were modeled as resistance elements (*R_B_*, *R_G_*). In addition, flow rate of both fluid was represented as *Q_B_* and *Q_G_*, respectively. As both fluids had the same pressure in coflowing channel, the following relation was derived as *P* = *R_B_* ∙ *Q_B_* = *R_G_* ∙ *Q_G_*. Here, a correction factor was suggested to compensate the boundary difference between real physical model and simple mathematical model. Based on the coflowing method with a correction factor [26], the blood viscosity (*μ_B_*) was expressed as $\mu_{B}=\mu_{G}\times CF\left(\alpha \right)\times \frac{\alpha }{1-\alpha }$. According to a previous study [27], a viscosity of 30% glycerin solution was provided as *μ_G_* = 2.73 ± 0.05 cP. In addition, based on a procedure discussed in a previous study [26], the correction factor of the coflowing method was estimated by analyzing the experimental results. As shown in Figure A1 (Appendix A), the correction factor (*CF(*α*)*) was obtained experimentally as *CF (α)* = −7.6315 *α^4^* +16.549 *α^3^* − 13.672 *α^2^* + 5.0285 α + 0.2652 (*R^2^* = 0.9626), which was validated within a range from *α* = 0.14 to *α* = 0.88. Under a stepwise varying blood sample flow rate, variations of *AI* and *μ**_B_* were monitored with respect to the shear rate.
